# Two Enteropathogenic *Escherichia coli* Strains Representing Novel Serotypes and Investigation of Their Roles in Adhesion

**DOI:** 10.4014/jmb.2105.05016

**Published:** 2021-07-15

**Authors:** Jing Wang, HongBo Jiao, XinFeng Zhang, YuanQing Zhang, Na Sun, Ying Yang, Yi Wei, Bin Hu, Xi Guo

**Affiliations:** 1 TEDA Institute of Biological Sciences and Biotechnology, Nankai University, 23 Hongda Street, TEDA, Tianjin 300457, P.R. China; 2 The Key Laboratory of Molecular Microbiology and Technology, Ministry of Education, 23 Hongda Street, TEDA, Tianjin 300457, P.R. China; 3 Shandong Center for Disease Control and Prevention, 16992 City Ten Road, Jinan 250014, Shandong, P.R. China; 4 LanLing Center for Disease Control and Prevention, 1 City Huibao Road, Lanling 276000, Lanling Shandong, P.R. China; 5 Taian Center for Disease Control and Prevention, 33 Changcheng Road, Taian 271000, Shandong, P.R. China; 6 Jinan KeJia Medical Laboratory, Inc., 800 Minghu West Road, Jinan 250001, Shandong, P.R. China

**Keywords:** Enteropathogenic *Escherichia coli*, O-antigen, O-antigen gene cluster, serotype, adhesion

## Abstract

Enteropathogenic *Escherichia coli* (EPEC), which belongs to the attaching and effacing diarrheagenic *E. coli* strains, is a major causative agent of life-threatening diarrhea in infants in developing countries. Most EPEC isolates correspond to certain O serotypes; however, many strains are nontypeable. Two EPEC strains, EPEC001 and EPEC080, which could not be serotyped during routine detection, were isolated. In this study, we conducted an in-depth characterization of their putative O-antigen gene clusters (O-AGCs) and also performed constructed mutagenesis of the O-AGCs for functional analysis of O-antigen (OAg) synthesis. Sequence analysis revealed that the occurrence of O-AGCs in EPEC001 and *E. coli* O132 may be mediated by recombination between them, and EPEC080 and *E. coli* O2/O50 might acquire each O-AGC from uncommon ancestors. We also indicated that OAgknockout bacteria were highly adhesive in vitro, except for the EPEC001 *wzy* derivative, whose adherent capability was less than that of its wild-type strain, providing direct evidence that OAg plays a key role in EPEC pathogenesis. Together, we identified two EPEC O serotypes in silico and experimentally, and we also studied the adherent capabilities of their OAgs, which highlighted the fundamental and pathogenic role of OAg in EPEC.

## Introduction

*Escherichia coli*, the predominant facultative anaerobe in human colonic flora, comprises both commensal and pathogenic strains, with the latter causing various diseases from gastroenteritis to extraintestinal infections [1]. To date, eight pathovars of *E. coli* have been reported, which can be broadly classified as diarrhoeagenic and extraintestinal *E. coli* (ExPEC) [2, 3]. Among the diarrheagenic *E. coli*, enteropathogenic *E. coli* (EPEC) is a major causative agent of life-threatening diarrhea in infants in developing countries [4].

Lipopolysaccharide (LPS), a component of the outer membrane, is located exclusively in the outermost layer of gram-negative bacteria. LPS typically consists of three components: lipid A, core oligosaccharides and O-antigen (OAg). The OAg is the most surface-exposed part of the LPS, and is usually a polymer comprising repeating oligosaccharide units (O-units), each containing two to eight sugar residues from a broad range of common or rare sugars and their derivatives [5].

The variability in OAgs is the basis for the serotyping systems of gram-negative bacteria. The antigenic scheme of *E. coli* was first presented by Fritz Kauffmann in the 1940s, and to date, more than 180 serotypes have been internationally recognized [6]. The genes for OAg synthesis are usually present at a specific locus on the chromosome, named the O-antigen gene cluster (O-AGC). These genes are normally classified into three groups: nucleotide sugar precursor synthesis genes (for biosynthesis of nucleotide sugar precursors specific to OAg), glycosyltransferase genes (for the sequential and specific addition of sugars that generate the O-units), and OAg processing genes (for OAg assembly) [7]. In *E. coli* strains, O-AGC usually maps between the housekeeping genes, *galF* and *gnd*, with a few exceptions, including O8/O9/O9a located between *gnd* and hisI genes [8, 9] and O62, which is located far from the *galF* and *gnd* loci [10].

Currently, two pathways have been identified as responsible for the assembly of *E. coli* OAgs: the Wzx/Wzy-dependent pathway and the ABC transporter (Wzm/Wzt)-dependent pathway. In each pathway, OAg synthesis is initiated by the transfer of sugar phosphate from NDP-sugar to undecaprenyl phosphate (Und-P) [11]. In the Wzx/Wzy pathway, other sugars are transferred sequentially to Und-PP-sugar to form O-units, which are then flipped by Wzx and polymerized by Wzy to generate the polymer [12]. In the Wzm/Wzt pathway, the sugars for each O-unit are sequentially added to Und-PP-sugar, designated as the “primer,” and the process does not stop until the complete repeating-unit polymer is generated, followed by the translocation of UndPP-OAg from the cytoplasm to periplasm by the ABC transporter [13].

During the pathogenesis, adhesion to epithelial cells is a key virulence function. EPEC adheres to the enterocytes in the small bowel and enables the colonization of intestinal epithelium, forming attaching and effacing (A/E) lesions and translocating effector proteins into host cell cytoplasm [14]. The majority of the genes required for A/E lesion formation are grouped within a pathogenicity island named the ‘locus of enterocyte effacement’ (LEE) [15]. Two LEE-encoded adhesins, type III secretion system (TTSS) EspA filaments and the outer-membrane adhesin, intimin (interacted with its translocated receptor Tir), have been reported possessing the ability to facilitate the adhesion of EPEC to intestinal epithelium [16]. In addition, the non-LEE-encoded factors, including the type IV bundle-forming pilus (BFP) and EspFu can also trigger EHPC adhesion [16, 17]. Several studies on other bacteria by comparing the wild-type strain with the OAg-deficient mutant provided evidence that OAg plays a key role in bacterial adhesion, thus affecting pathogenesis [18-20], as well as enabling the bacteria to evade the host immune system [21, 22]. However, the adherent and pathogenic role of OAg in EPEC is still largely unknown. The aim of this study was to characterize the putative novel O-AGC loci of two EPEC strains isolated from Shandong Province, China, during routine detection. Moreover, mutagenesis of the O-AGCs was constructed and used for functional analysis of the loci, and the roles of the OAgs of these two strains in virulence were also investigated via in vitro experiments.

## Materials and Methods

### Bacterial Strains, Plasmids, and Growth Conditions

The two EPEC strains, EPEC001 from a patient's fecal sample, and EPEC080 from a goat were isolated by the Shandong Center for Disease Control and Prevention. Details of EPEC001, EPEC080, their derivatives, and plasmids are described in Table 1. The primers used for mutant construction are also listed in Table 1. All strains were routinely cultured in 2× YT medium (16 g tryptone, 10 g yeast extract, and 5 g sodium chloride per liter). When necessary, the media were supplemented with chloramphenicol (Cm, 25 μg/ml) or blasticidin (Bs, 200 μg/ml).

### Genome Sequencing, Assembly, and Annotation

Genomic DNA was extracted from 1.5 ml of overnight bacterial culture (approximately 10^8^ colony-forming units (CFU)/ml) using a DNA extraction kit (Tiangen, China) according to manufacturer's instructions. Subsequently, the DNA was sheared, polished, and prepared using the Illumina Sample Preparation Kit. Genome sequencing was performed using the Solexa sequencing technology (Illumina Inc., USA) and the reads obtained were assembled using the de novo genome-assembly program Velvet to generate a multi-contig draft genome. Artemis [23] was used to annotate genes, and the lockMaker program [24] was used to identify conserved motifs. BLAST and PSI-BLAST [25] were used to search genes and proteins against the available databases including GenBank (www.ncbi.nlm.nih.gov/genbank) and Pfam protein families database (pfam.sanger.ac.uk). TMHMM v2.0 (http://www.cbs.dtu.dk/services/TMHMM-2.0/) was used to identify potential transmembrane domains within protein sequences. The putative O-AGC between the *galF* and *gnd* genes of each strain was retrieved from the genomes for further analysis.

### Construction of Mutants

The mutant strains were constructed using a λ Red recombinase system as previously described[26]. Briefly, first, the plasmid pSim17 was electroporated into the wild-type (WT) strain to enable a direct homologous recombination with PCR products. Following this, the chloramphenicol acetyltransferase (*cat*) gene from plasmid pKD3 was amplified using 50 nucleotides homologous to the flanking regions of the DNA target segment and the PCR product was transformed into the pSim17-containing wild-type strain that could express recombinase. The mutants with the introduced *cat* gene were confirmed by PCR and sequencing.

### LPS Preparation and Analysis

LPS was extracted using the hot aqueous-phenol method as previously described [27]. The extracted LPSs were separated by using 12% SDS-PAGE at 50 V for 30 min and 100 V for 2 h and subsequently, they were visualized by silver staining using the Fast Silver Stain Kit (No. P0017S, Beyotime, China) according to manufacturer’s protocol. The gel image was captured using a GS900 Calibrated Densitometer (BioRad Laboratories, USA) under “silver stain” mode.

### Cell Culture and Bacterial Adhesion

HeLa cells were cultured in high-glucose Dulbeccós modified Eaglés medium containing 10% fetal bovine serum and penicillin-streptomycin-glutamine and they were grown at 37°C under 5% CO_2_. For adhesion assays, cells grown overnight to approximately 80% confluence were seeded into 12-well tissue culture plates at a concentration of 1×10^6^ cells per well and they were maintained as differentiated monolayers. Next, bacteria in the logarithmic growth phase were added to the cell monolayers at an MOI of of 10. After 6 h of incubation at 37°C, the cells were washed extensively with phosphate-buffered saline (PBS) three times to remove non-adherent bacteria and they were permeabilized with 0.2% Triton X-100. The adhesive bacteria were collected, serially diluted in PBS, and spread onto Luria-Bertani agar for counting bacterial CFUs. Three independent experiments were performed for each strain. Statistical significance was determined using an unpaired Student's *t*-test. A p-value of < 0.05 was considered statistically significant.

### Nucleotide Sequence Accession Number

The DNA sequences of the O-AGCs from EPEC001 and EPEC080 were deposited in GenBank database under accession numbers MW690110 and MW690111, respectively.

## Results

### Functional Annotation of Putative O-AGCs

**Fig. 1:**
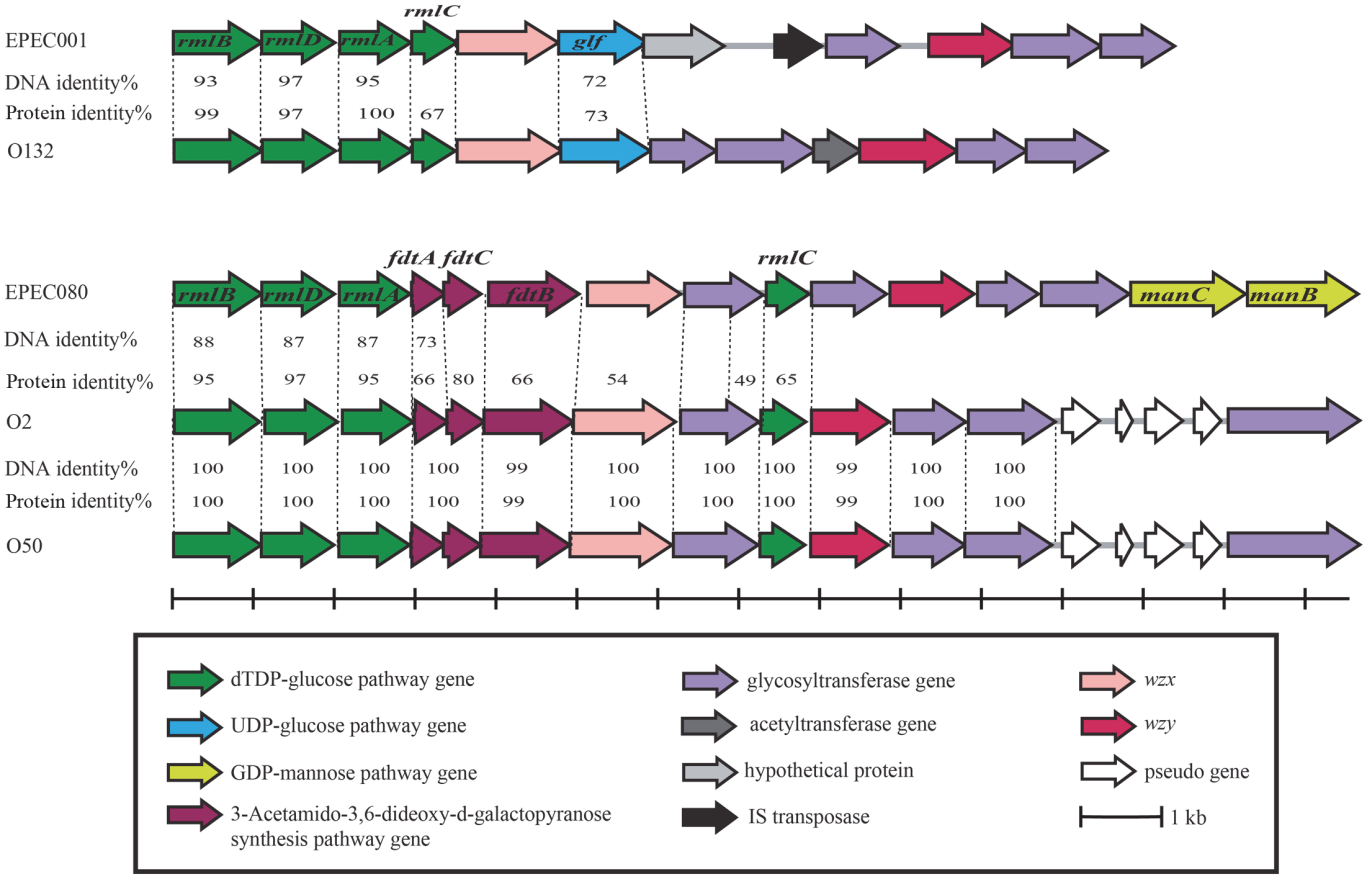
O-antigen gene clusters of EPEC001 and EPEC080, and comparisons with each related serotype(s). *rmlA*, glucose-1-phosphate thymidylyltransferase gene; *rmlB*, dTDP-D-glucose 4,6-dehydratase gene; *rmlC*, dTDP-4-keto-6- deoxy-D-glucose 3,5-epimerase gene; *rmlD*, dTDP-6-deoxy-L-mannose-dehydrogenase gene; *glf*, UDP-galactopyranose mutase gene; *fdtA*, dTDP-6-deoxy-hex-4-ulose isomerase gene; *fdtB*, dTDP-6-deoxy-D-xylo-hex-3-ulose aminase gene; *fdtC*, dTDP-D-Fuc3N acetylase gene; *manB*, phosphomannomutase gene; *manC*, mannose-1-phosphate guanylyltransferase gene; *wzx*, O-antigen flippase gene; *wzy*, O-antigen polymerase gene.

The putative O-AGC of EPEC001 is 12,344 bp in length, and it contains 12 open reading frames (*orfs*) with the same transcriptional direction from *galF* to *gnd* (Fig. 1, Table 2). *orf1* to *4* are identical to the dTDP-glucose 4,6-dehydratase (*rmlB*), dTDP-4-dehydrorhamnose reductase (*rmlD*), glucose-1-phosphate thymidylyltransferase (*rmlA*), and dTDP-4-dehydrorhamnose 3,5-epimerase (*rmlC*) genes, respectively. A set of genes in the order *rmlBDAC* is usually localized at the 5’ end of O-AGC and the products are responsible for the biosynthesis of dTDP-L-Rha, the nucleotide precursor of L-Rha, which is usually found as a component of OAg. *orf6* was assigned as *glf*, which encodes a UDP-galactopyranose mutase catalyzing the formation of UDP-D-Gal*f*, which is the nucleotide precursor of D-Gal*f*, from UDP-D-Gal. *orf5* and *orf10* were presumptively identified as *wzx* and *wzy*, respectively, using BLAST. Additionally, Orf5 contains 11 predicted transmembrane (TM) domains, which is the typical number for OAg flippase (Wzx), and Orf10 contains nine predicted TM domains, which is typical for OAg polymerase (Wzy). *orf9*, *orf11*, and *orf12* were all assigned as glycosyltransferase genes. Moreover, *orf9* was predicted as the rhamnosyltransferase gene and *orf12* as the galactofuranosyltransferase gene, which suggests that the occurrence of L-Rha and Galf residues in EPEC001 OAg is very likely and is in accordance with the existence of *rmlBDAC* (*orf1* to 4) and *glf* (*orf6*) genes. Among the remaining two *orfs*, the product of *orf8* was predicted to be the ISAs1 family transposase and the product of *orf7* exhibited no similarity to any functionally characterized/identified protein, and therefore, it was assigned as a hypothetical protein.

The putative O-AGC of EPEC080 also maps between *galF* and *gnd* genes, with 14,550 bp in length and 15 *orfs* being annotated (Fig. 1, Table 2). *orf1* to *3* and *orf9* were annotated as *rmlB*, *rmlD*, *rmlA*, and *rmlC*, respectively. *orf4* to *6*, the three *orfs* downstream of *rmlA*, were assigned to the isomerase (*fdtA*), N-acetyltransferase (*fdtC*), and transaminase (*fdtB*) genes, respectively. The products of these three genes along with RmlA and RmlB are responsible for the synthesis of dTDP-D-Fuc3NAc from Glc-1-P. dTDP-D-Fuc3NAc is the nucleotide precursor of D-Fuc3NAc, which is a rare sugar occasionally occurring in OAg. *orf14* and *orf15* were assigned as the mannose-1-phosphate guanylyltransferase (*manC*) and phosphomannomutase (*manB*) genes, respectively. ManB, ManC, and the phosphomannose isomerase, ManA, are involved in the synthesis of GDP-D-Man, the nucleotide precursor of D-Man, from Fru-6-P. However, the *manA* gene is not always located in O-AGC. *orf7* and *orf11* were functionally annotated as *wzx* and *wzy*, respectively. TM domain analysis revealed that Orf7 contained 10 TM segments and Orf11 contained eight TM segments, each exhibiting typical features of O-antigen flippase (Wzx) and O-antigen polymerase (Wzy). The remaining four *orfs* were predicted to encode glycosyltransferases. Furthermore, among them, *orf10*, *11*, and *13* were assigned to the rhamnosyltransferase, glucosyltransferase, and mannosyltransferase genes, respectively. This indicates the existence of L-Rha, D-Glc, and D-Man residues in EPEC080 OAg, and it partly verifies the gene annotation of O-AGC.

### Construction of Mutant Strains

**Fig. 2:**
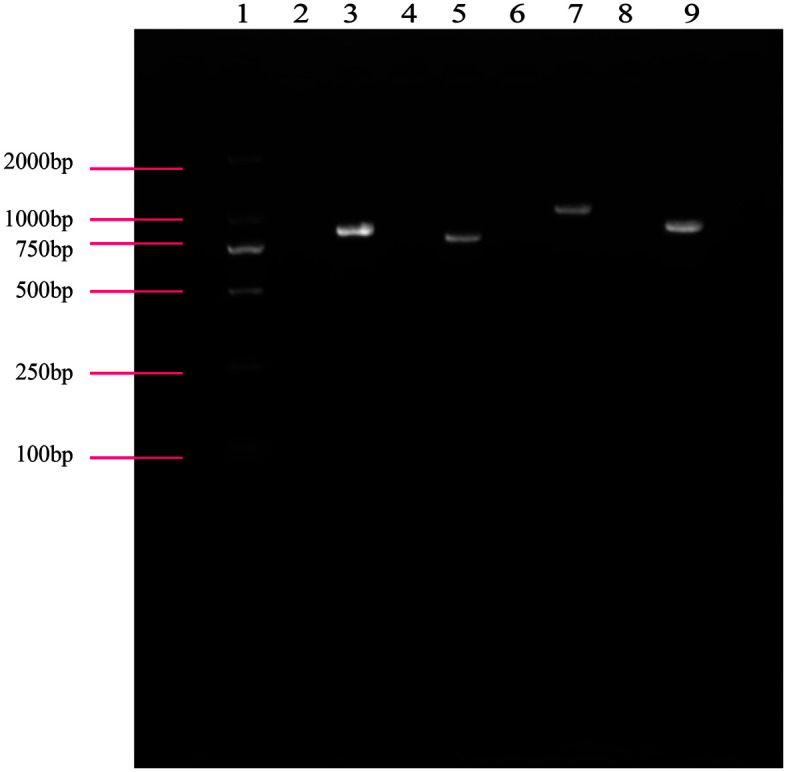
Agarose gel electrophoresis of all PCR products from the mutant strains and their wild-type controls. Lane 1: DL2000 DNA marker; lane 2: EPEC001 (Vcat/VOAg001); lane 3: EPEC001ΔOAg (Vcat/VOAg001); lane 4: EPEC001 (Vcat/Vwzy001); lane 5: EPEC001Δ*wzy* (Vcat/Vwzy001); lane 6: EPEC080 (Vcat/VOAg080); lane 7: EPEC080ΔOAg (Vcat/ VOAg080); lane 8: EPEC080 (Vcat/Vwzy080); lane 9: EPEC080Δ*wzy* (Vcat/Vwzy080).

To determine the functional roles of putative O-AGCs, mutant strains with O-AGC and *wzy* deletions were constructed for EPEC001 and EPEC080, respectively, and verified by PCR amplification and subsequent sequencing. For PCR, the common forward primer was designed in the *cat* gene of pKD3 that substitutes the DNA segment of the deleted gene(s) and the reverse primer was designed in the *orf* downstream of the targeted gene(s)(**Table 1**). As shown in the agarose gel electrophoresis of all PCR products (Fig. 2), each mutant strain generated a specific and length-correct band, with the corresponding wild-type strain (control) giving no PCR product. All constructed mutant strains were further confirmed by sequencing.

### Functional Confirmation of O-AGCs

**Fig. 3:**
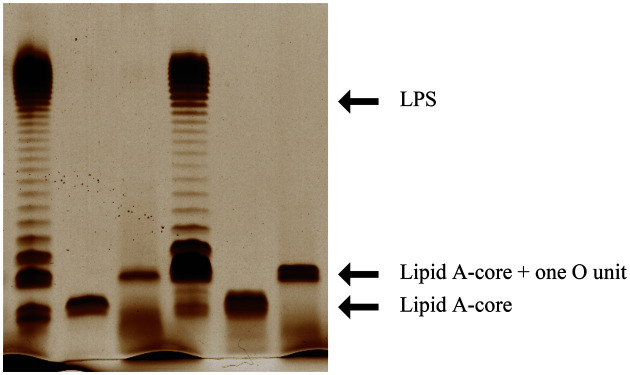
Lipopolysaccharide profiles of strains EPEC001, EPEC080, and their derivatives. The extracts were electrophoresed on 12% SDS-PAGE and stained by silver staining. From left to right: EPEC001 expressing complete LPS, EPEC001ΔOAg expressing rough LPS, EPEC001Δ*wzy* expressing semi-rough LPS, EPEC080 expressing complete LPS, EPEC080ΔOAg expressing rough LPS, and EPEC080Δ*wzy* expressing semi-rough LPS.

As shown in the LPS profile (Fig. 3), EPEC001 generated a WT bimodal distribution of LPS, characterized by a first band of lipid A-core and additional bands corresponding to O-units. However, the mutant EPEC001ΔOAg only generated one band of lipid A-core and no attached OAg, and the mutant EPEC001Δ*wzy* showed a semi-rough LPS phenotype with only one O-unit substitution on the lipid A-core. These results collectively indicate that the locus between *galF* and *gnd* is effectively involved in the biosynthesis of EPEC001 OAg and that *orf10* encodes the O-antigen polymerase (Wzy) responsible for the OAg assembly in EPEC001. Similar to EPEC001 and its derivatives, EPEC080 exhibited a complete LPS profile, while the EPEC080ΔOAg strain showed a rough LPS phenotype and the EPEC080Δ*wzy* strain showed a semi-rough LPS phenotype. Thus, we demonstrated that the O-AGC of EPEC080 maps between *galF* and *gnd*, and the *wzy* (*orf11*) gene of the strain is involved in OAg assembly.

### The Role of OAgs in Adhesion

**Fig. 4:**
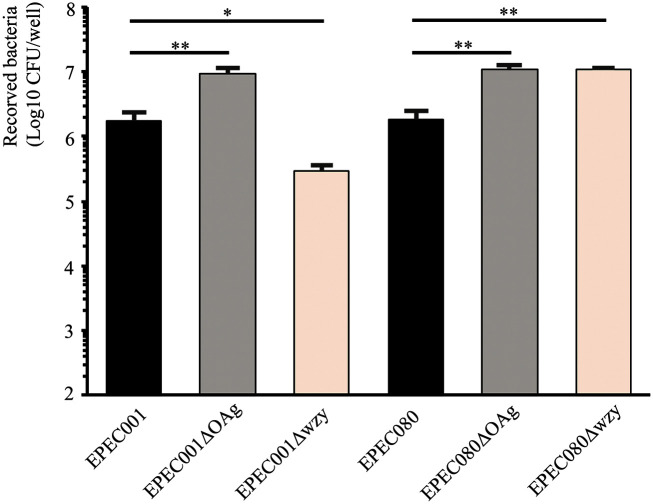
Adherent capabilities of EPEC001, EPEC080, and their derivatives. Data are presented as means ± standard deviations (SD) for three biological replicates. Statistical analysis was performed using the unpaired Student *t*-test. *p < 0.05, **p < 0.01.

Except for the enteroinvasive *E. coli*, adhesion to host cells is a requirement for all E.coli pathovars and it is a key stage in bacterial infection. To investigate the pathogenic role of OAg, we assessed its adhesion ability to the HeLa cells of EPEC001, EPEC080, and their OAg derivatives (Fig. 4). After 6 h of infection, compared to the EPEC080 WT strain, the mutant complete loss of O-AGC and the mutant loss of *wzy* both exhibited increased bacterial adhesion (6-fold, *p* = 0.0067 and 5.9-fold, *p* = 0.0061, respectively). For EPEC001, the adhesion ability of its O-AGC mutant also significantly increased as compared to that of the WT strain (5.9-fold, *p* = 0.0093). However, the *wzy* mutant generated a completely opposite result, and the loss of *wzy* significantly decreased the bacterial adhesion level (0.16-fold, *p* = 0.014).

## Discussion

In this study, we genetically characterized novel putative O-AGCs from two *E. coli* strains that could not be tested using Iguchi's O-genotyping PCR assay, which targets almost all known *E. coli* serotypes [28]. To date, more than 180 O serotypes of *E. coli* strains have been identified based on their OAg variability. Several *E. coli* O serotypes are closely associated with human diseases with high morbidity and mortality rates. For example, the Shiga toxin-producing *E. coli* (STEC) O157:H7 is a well-known pathogenic clone that causes hemolytic-uremic syndrome and foodborne illnesses [29]. The “big six” non-O157 STEC strains (O_2_6, O_4_5, O103, O111, O121, and O145) cause less severe infections than O157. However, the severity of infection differs with different serotypes. Another example is the STEC O104:H4, which caused a widespread and severe foodborne illness epidemic in Germany in 2011 [30]. In recent years, strains representing novel serotypes and being associated with or as one of the dominant clones causing human diseases have been isolated and characterized [31-33]. Pair-to-pair alignment shows that the O-AGC of EPEC001 is close to that of *E. coli* O132 and the O-AGC of EPEC080 is related to that of *E. coli* O2/O50 (Fig. 1). Generally, products encoded by the six upstream genes including *wzx* of EPEC001 O-AGC, share high percentages of protein identity levels (50 to 100) to the corresponding region of *E. coli* O132 O-AGC with the right region being unique, which is probably the serotype determinant. Indeed, all glycosyltransferase genes and *wzy*, the possessing gene, which are considered highly sero-specific, were located at the 3' end of O-AGC. It seems that a recombination event occurred between the O-AGCs of EPEC001 and *E. coli* O132 and we propose that one of the recombination sites is located in the *rmlA* gene since the DNA identity level of *rmlA* was much higher than that of its downstream genes. Likewise, the first nine genes in the O-AGCs of EPEC080 and *E. coli* O2/O50 have the same order and they share 49 to 95% protein identity level. However, the DNA identity level of each gene pair is less than 90%. Thus, it is proposed that both EPEC080 and *E. coli* O2/O50 have each acquired their respective O-AGC from uncommon ancestors instead of via recombination between them. As the diversity of serotypes is based on the structural variations of OAgs and O-AGC is usually closely related to OAg, an elucidation of OAg structures needs to be conducted in the future to provide an insight into the evolution of the O-AGCs of novel serotypes.

The presence and length of OAgs play a key role in bacterial pathogenesis, and they protect the bacteria by evading the host innate immune response. ExPEC strains such as UPEC and NMEC are always triggered to be resistant to host systemic immunity by expressing specific surface polysaccharides, mainly including a capsule and/or OAg [21, 22, 34]. While the host-diarrheagenic *E. coli* interactions are primarily mediated by bacterial invasive virulence factors, the role of surface polysaccharides in the pathogenesis and regulation of intestinal inflammation remains unclear. Recently, it has been reported that the OAg of adherent-invasive *E. coli* (AIEC) can reduce its ability to adhere to and invade intestinal epithelial cells in vitro and regulate host inflammation via complement C3 [35]. EPEC can be further divided into “typical” and “atypical” subtypes based on the presence or absence of *E. coli* adherence factor plasmid [36] with each subtype containing frequently isolated serotypes or being non-typeable [14]. To the best of our knowledge, whether OAg affects the adhesion to and invasion into the intestinal epithelial cells of EPEC is rarely studied. A much earlier study reported that an EPEC strain, B171 (serotype O111), exhibits localized adherence to HeLa cells and this process is mediated by the OAg encoded by plasmid pYR111 [37]. In the present study, we investigated the role of OAg in the adhesion of EPEC to HeLa cells. The increased adhesion abilities of EPEC001ΔOAg and EPEC008 OAg derivatives are very similar to those observed in *Shigella sonnei* [38], *Salmonella enterica* [39], and *Burkholderia cenocepacia* [40]. All these findings suggest that OAg may mask one or more bacterial surface adhesins and OAg expression modulates bacterial pathogenesis by balancing their adherent and invasive capabilities and conferring optimal protection against host defense mechanisms. However, EPEC001Δ*wzy* generates an opposite result, that is, this OAg isogenic strain attenuated the adherent ability to HeLa cells, similar to that observed in AIEC [35] and *Plesiomonas shigelloides* [41]. Another report also revealed that the deletion of the *wzy* gene significantly decreases the adherent and invasive abilities of avian pathogenic *E. coli* [42]. Thus, contrasting conclusions exist on the pathogenesis of OAgs in different species and strains. We propose that on one hand, this is attributed to the different chemical structures of OAgs. On the other hand, OAg may indirectly contribute to pathogenesis, that is, its expression may be regulated by various upstream signals and other virulence factors can also be adjusted by OAg. Therefore, the OAg-associated regulatory pathway of pathogens needs to be further characterized individually.

In general, we characterized and identified two EPEC serotypes in silico and experimentally, thus further expanding the current *E. coli* serotyping scheme. We also evaluated the in vitro adherent capabilities of the OAgs of two strains, and our findings highlight the fundamental and pathogenic role of OAg in EPEC.

## Figures and Tables

**Table 1:** Strains, plasmids, and primers.

Strain/plasmid/primer	Description
Bacterial strain	
EPEC001	Wild-type strain
EPEC001ΔOAg	Deletion of the entire O-antigen gene cluster, Cm^r^
EPEC001Δ*wzy*	Deletion of the *wzy* gene, Cm^r^
EPEC080	Wild-type strain
EPEC080ΔOAg	Deletion of O-antigen gene cluster, Cm^r^
EPEC080Δ*wzy*	Deletion of the *wzy* gene, Cm^r^
Plasmid	
pSim17	Plasmid carrying genes encoding lambda Red recombinase system, Bs^r^
pKD3	Template for PCR amplification of Red recombinase-medicated recombination, Cm^r^
Primer	Nucleotide sequences (5'-3') ^a^
FOAg001	**ACATTTATTGAAACCAATATTGTTGGTACTTATGTCCTTTTGGAAGCCGC**CATATGAATATCCTCCTTAG, forward primer for EPEC001 O-antigen gene cluster deletion
ROAg001	**TATAAGCATCAAAACATATCCTAGCGGCTTTTACATTTCCAGTTAACATT**GTGTAGGCTGGAGCTGCTTCG, reverse primer for EPEC001 O-antigen gene cluster deletion
Fwzy001	**TTATTAAATATATGTTTATCAAGGCTTTCTACAAATCCTTTGATTTTATT**CATATGAATATCCTCCTTAG, forward primer for EPEC001 *wzy* gene deletion
Rwzy001	**ATCATTCCATAATATTAACCATATTAATGATACTACATAAGTATTAAAAG**GTGTAGGCTGGAGCTGCTTCG, reverse primer for EPEC001 *wzy* gene deletion
FOAg080	**CTGGCTGCTGAAAGCCATGTGGATCGTTCCATTACAGGCCCTGCGGCATT**CATATGAATATCCTCCTTAG, forward primer for EPEC080 O-antigen gene cluster deletion
ROAg080	**CTACCAGCAGCCACGGGATCATGCCGCTGTCGCAGTAAGCGAAATCACGG**GTGTAGGCTGGAGCTGCTTCG, reverse primer for EPEC080 O-antigen gene cluster deletion
Fwzy080	**TTGGCTTTTGCGTTTATTTCTATTTATTACAAGGCTAAGGCAATAAGGCT**CATATGAATATCCTCCTTAG, forward primer for EPEC080 *wzy* gene deletion
Rwzy080	**TAATTCACTCATGCGCAAAGAAAATGCTGGCACAAACGAAAATAAAACTA**GTGTAGGCTGGAGCTGCTTCG, reverse primer for EPEC080 *wzy* gene deletion
Vcat	ATGGACAACTTCTTCGCC, forward primer for each mutant verification, designed in *cat* gene of pKD3
VOAg001	ACGAGGCGTTTCAAGAGA, reverse primer for EPEC001ΔOAg verification, designed in *gnd* of EPEC001, 856bp
Vwzy001	GTTGGAAATAAATGGCTGTG, reverse primer for EPEC001Δ*wzy* verification, designed in *orf11* of EPEC001 O-AGC, 774bp
VOAg080	ACTAACCACTGGACTTGCTC, reverse primer for EPEC080ΔOAg verification, designed in *gnd* of EPEC080, 1002bp
Vwzy080	CCACTGTTGGCTTTTGTTT, reverse primer for EPEC080Δ*wzy* verification, designed in *orf12* of EPEC080 O-AGC, 867bp

^a^Boldface characters indicate the 50 nucleotides homologous to the initial and final portions of the target DNA segment.

**Table 2:** Characteristics of open reading frames (ORFs) in the O-antigen gene clusters of EPEC001 and EPEC080.

Orf no.	Gene name	Position of the gene	G+C content (%)	Similar protein(s), strain(s) (GenBank accession no.)	%Identical/%Similar (total no. of aa)	Putative function of protein
EPEC001						
1	*rmlB*	1..1086	42.44	dTDP-glucose 4,6-dehydratase [*Escherichia coli*] (WP_052925278.1)	100/100 (361)	dTDP-glucose 4,6-dehydratase
2	*rmlD*	1086..1985	47.55	dTDP-4-dehydrorhamnose reductase [*Escherichia coli*] (WP_046201417.1)	99/100 (299)	dTDP-4-dehydrorhamnose reductase
3	*rmlA*	2043..2918	43.26	glucose-1-phosphate thymidylyltransferase RfbA [*Escherichia coli*] (WP_046201417.1)	99/100 (291)	Glucose-1-phosphate thymidylyltransferase
4	*rmlC*	2927..3481	32.43	dTDP-4-dehydrorhamnose 3,5-epimerase [*Escherichia coli*] (WP_057080958.1)	99/100 (184)	dTDP-4-dehydrorhamnose 3,5-epimerase
5	*wzx*	3490..4728	34.22	O34 family O-antigen flippase [*Escherichia coli*] (WP_097479960.1)	51/71 (412)	flippase
6	*glf*	4731..5822	32.6	UDP-galactopyranose mutase [*Escherichia coli*] (WP_033560995.1)	75/85 (363)	UDP-galactopyranose mutase
7		5825..6814	32.12	hypothetical protein [*Escherichia coli*] (WP_053273170.1)	99/99 (329)	hypothetical protein
8		7459..8013	40.24	ISAs1 family transposase [*Escherichia coli*] (MBJ0238419.1)	96/97 (184)	H repeat-associated protein
9		8068..8961	32.66	glycosyltransferase [*Escherichia coli*] (WP_085446706.1)	38/59(297)	glycosyltransferase family 2 protein
10	*wzy*	9274..10335	29.75	EpsG family protein [*Cronobacter muytjensii*] (WP_075192411.1)	47/69 (353)	polymerase
11		10345..11439	29.22	glycosyltransferase family 4 protein [*Cronobacter muytjensii*] (WP_083605367.1)	46/64 (364)	glycosyltransferase
12		11436..12344	31.35	glycosyltransferase family 2 protein [*Enterobacter asburiae*] (WP_150182824.1)	62/77 (303)	Galactofuranosyltransferase GlfT1
EPEC080						
1	*rmlB*	1..1086	43.18	dTDP-glucose 4,6-dehydratase [*Escherichia coli*] (WP_029399178.1)	100/100 (361)	dTDP-glucose 4,6-dehydratase
2	*rmlD*	1086..1985	46.11	dTDP-4-dehydrorhamnose reductase [*Escherichia coli*] (WP_029399176.1)	100/100（299）	dTDP-4-dehydrorhamnose reductase
3	*rmlA*	2043..2921	43.34	glucose-1-phosphate thymidylyltransferase RfbA [*Escherichia coli*] (WP_029399175.1)	100/100 (292)	Glucose-1-phosphate thymidylyltransferase 1
4	*fdtA*	2935..3354	32.85	FdtA/QdtA family cupin domain-containing protein [*Cedecea lapagei*] (WP_126355658.1)	66/83(139)	TDP-4-oxo-6-deoxy-alpha-D-glucose-3,4-oxoisomerase
5	*fdtC*	3332..3796	36.77	N-acetyltransferase [*Escherichia coli*] (EFN7827253.1)	100/100（154）	dTDP-3-amino-3,6-dideoxy-alpha-D-galactopyranose 3-N-acetyltransferase
6	*fdtB*	3801..4922	33.77	DegT/DnrJ/EryC1/StrS family aminotransferase [*Escherichia coli*] (HAO2821289.1)	99/99（373）	dTDP-3-amino-3,6-dideoxy-alpha-D-galactopyranose transaminase
7	*wzx*	4906..6177	30.47	O50 family O-antigen flippase [*Escherichia coli*] (EFN5080582.1)	54/74（423）	flippase
8		6190..7221	31.2	glycosyltransferase family 4 protein [*Enterobacter cloacae* complex sp.] (WP_133294767.1)	53/71（343）	glycosyltransferase family 4 protein
9	*rmlC*	7234..7767	34.08	dTDP-4-dehydrorhamnose 3,5-epimerase [*Escherichia coli*] (EEW2230532.1)	99/100（177）	dTDP-4-dehydrorhamnose 3,5-epimerase
10		7793..8722	30.96	glycosyltransferase family 2 protein [*Escherichia coli*] (WP_063610376.1)	48/69（309）	rhamnosyltransferase
11	*wzy*	8762..9805	27.2	EpsG family protein [*Escherichia coli*] (WP_089723541.1)	45/66（347	polymerase
12		9844..10599	28.04	glycosyl transferase group 2 family protein [*Escherichia coli*] (OAC41241.1)	58/76（251)	UDP-Glc:alpha-D-GlcNAc-diphosphoundecaprenol beta-1,3-glucosyltransferase WfgD
13		10613..11722	30.21	glycosyltransferase [*Croceivirga radicis*] (WP_080317782.1)	52/71（369）	Phosphatidyl-myo-inositol mannosyltransferase
14	*manC*	11738..13159	36.42	mannose-1-phosphate guanylyltransferase/mannose-6-phosphate isomerase [*Escherichia coli*](WP_029399160.1)	100/100(473)	Mannose-1-phosphate guanylyltransferase 1
15	*manB*	13180..14550	54.48	phosphomannomutase/phosphoglucomutase [*Escherichia coli*] (EFA9345916.1)	99/99(456)	Phosphomannomutase/phosphoglucomutase
